# Supplementary figures and images for: Critically ill COVID-19 patients with neutralizing autoantibodies against type I interferons have increased risk of herpesvirus disease
Source: PLoS Biol. 2022 Jul 5;20(7):e3001709. doi: 10.1371/journal.pbio.3001709 (PMC9286229; doi:10.1371/journal.pbio.3001709)

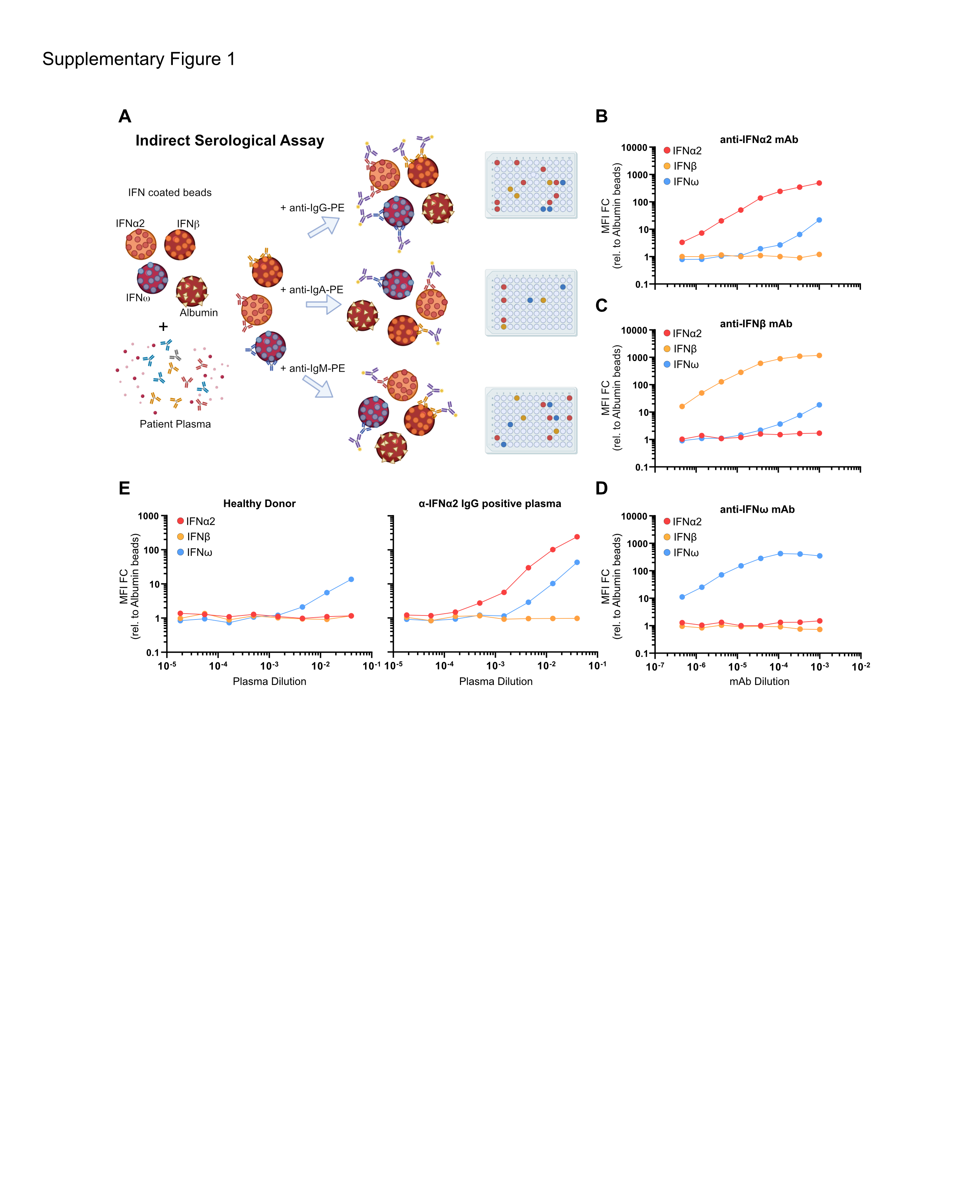

Supplement: S1 Fig — (A) Schematic representation of the assay principle. Magnetic beads are covalently coated with the indicated IFNs or albumin as a negative control. Samples are then incubated with the coated beads for 1 h at room temperature to allow binding of any anti-IFN antibodies present. Following wash steps, PE-labeled secondary antibodies specific for antibody isotypes of interest (IgG, IgA, or IgM) are incubated with the beads. After washing, MFI values of bound PE secondary antibodies are measured for each “bead region” on a FlexMap 3D instrument. Schematic created with BioRender.com. (B, C, and D) Assay assessment using mouse monoclonal antibodies. IFNα2, IFNβ, IFNω, and albumin-coated beads mixed 1:1:1:1 were incubated with serial dilutions of mouse monoclonal antibodies raised against IFNα2 (B), IFNβ (C), or IFNω (D). Following the assay procedure described in (A), MFI values from IFN-coated beads were obtained and calculated relative to MFI values from albumin-coated beads. Data are representative of at least 2 independent experiments. (E) Assay assessment using human plasma samples. IFNα2, IFNβ, IFNω, and albumin-coated beads mixed 1:1:1:1 were incubated with serial dilutions of a pool of healthy donor plasmas (left panel) or a human plasma known to have anti-IFNα2 antibodies (right panel). Following the assay procedure described in (A), MFI values from IFN-coated beads were obtained and calculated relative to MFI values from albumin-coated beads. Data are representative of at least 2 independent experiments. Data underlying this figure can be found in S1 Data. FC, fold change; IFN, interferon; MFI, median fluorescence intensity; PE, phycoerythrin. (TIF) [file pbio.3001709.s001.tif]

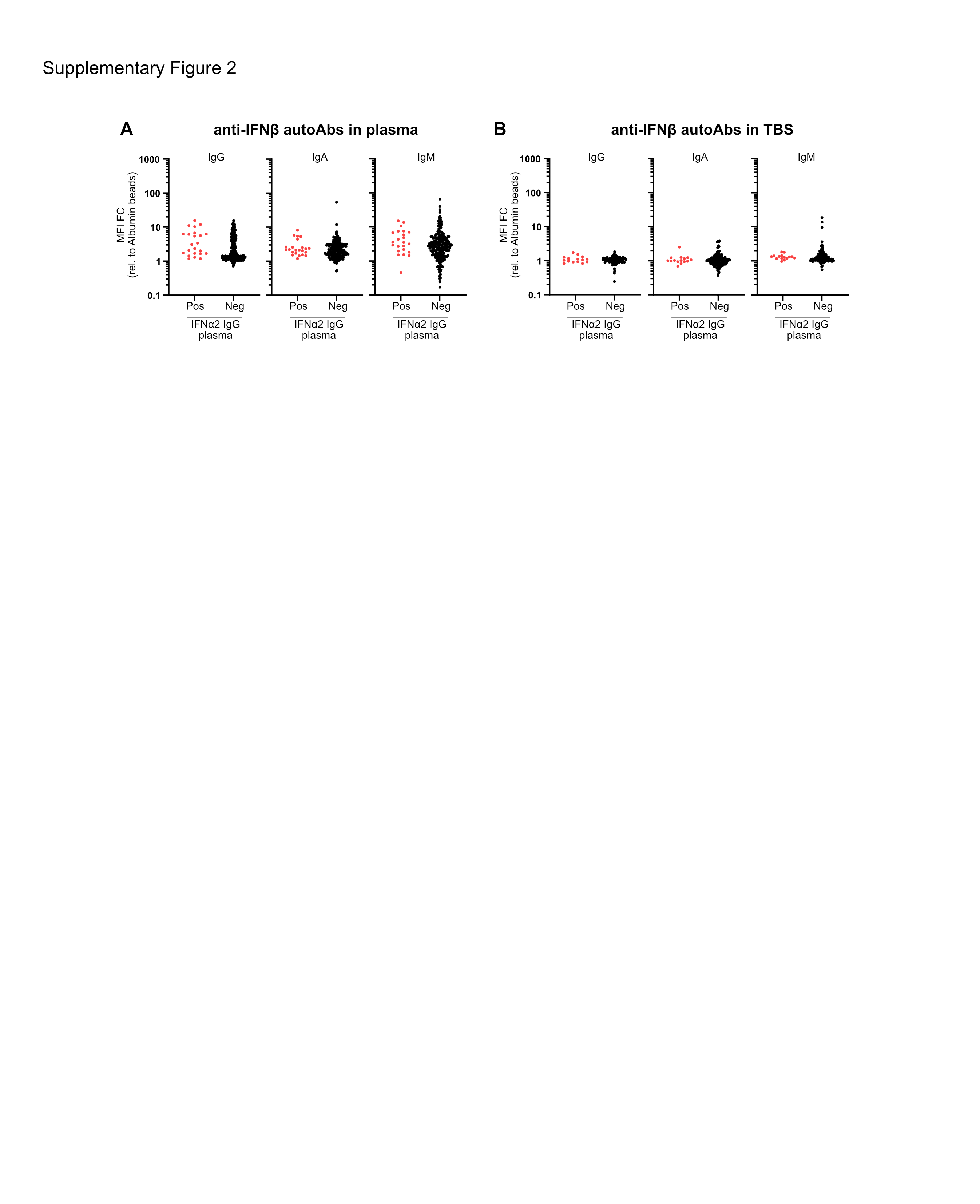

Supplement: S2 Fig — Multiplexed bead-based assay to detect IgG, IgA, and IgM autoantibodies (autoAbs) against IFNβ in the plasmas (A) or TBSs of COVID-19 ICU patients described in Fig 1A. Pos (positivity) and Neg (negativity) for anti-IFNα2 IgG in plasma samples from the same patient (results from Fig 1A) were used to stratify patients. MFI FC of signal derived from IFN-coated beads relative to the MFI of signal derived from albumin-coated beads is shown. In all panels, red dots indicate the patients/samples that were positive for anti-IFNα2 IgG autoantibodies in plasma (Fig 1A) and are denoted simply for reference. Data underlying this figure can be found in S1 Data. COVID-19, Coronavirus Disease 2019; FC, fold change; ICU, intensive care unit; IFN, interferon; MFI, median fluorescence intensity; TBS, tracheobronchial secretion. (TIF) [file pbio.3001709.s002.tif]
