# Supplementary material for: Critically ill COVID-19 patients with neutralizing autoantibodies against type I interferons have increased risk of herpesvirus disease
Source: PLoS Biol. 2022 Jul 5;20(7):e3001709. doi: 10.1371/journal.pbio.3001709 (PMC9286229; doi:10.1371/journal.pbio.3001709)
Supplement: Attachment — Submitted filename: 2_IFNautoAbs_ResponsetoReviewers_revision.docx [file pbio.3001709.s004.docx]

**Author response letter for PBIOLOGY-D-22-00687R1**

*We thank all the reviewers and the editors for their very positive assessment of our manuscript and for their highly constructive comments. Please find below all the points raised, with our responses indicated in blue italics.*

**Reviewer #1:**

This paper is excellent. Novel, important, and timely. Suggestions to improve it include:

1/ It is not clear if the entire cohort was tested for neutralization of IFN-alpha and -omega at low and high concentrations (as should be) or only the ELISA-positive patients. It is also not clear either if IFN-beta neutralization was tested.

*> We thank the reviewer for their very positive assessment of our study, including its novelty and importance. We have now made it clear in the main text which patient samples were tested for neutralisation, and commented on the importance of neutralisation assays to increase sensitivity and thereby detection of functionally-relevant autoAbs (lines 303-308).*

2/ They should quote all recent studies of auto-Abs to IFN in COVID patients, including Moscow, Japan, Bogota, and Leuven.

*> We have now updated our citations to include these latest studies, PMIDs: 34960822 (Moscow),* 35576468, **35291303** (Japan), **35296346** (Bogota), and 35296990 (Leuven) *(lines 68, 245, and 391).*

3/ They could cite the seminal 1984 paper Ion Gresser, especially because the viral disease was shingles.

*> We have now updated our citations to include this original study and have commented on its relation to our work (lines 405-406 and 451).*

4/ Describing the age of AAB+ patients in the COVID cohort in the text would be reader-friendly (it's in the table).

*> We have now added patient age information to lines 219 and 315-316.*

5/ For the HSV/CMV reactivation, a missing info is if the infection had any clinical impact. Was it CMV disease (GI involvement, eye, neuro etc.) or just positive PCR in blood? And if only PCR, which viral load?

*> This is an important point, and we have now added information relating to the clinical manifestations observed and PCR viral loads on lines 329-330 and 342-344.*

6/ About the reactivation of CMV and HSV, it is not entirely clear if those tests were performed only in patients who had positive serologies for those viruses. The term reactivation is used, implying that these patients were infected before.

*> This is an important point. PCRs were performed upon the treating physician’s decision and in most cases independent of previous knowledge of the serostatus of the individual. Some, but not all, individuals in the whole cohort were tested by serology for HSV or CMV IgG and IgM. Of these individuals, only one CMV primoinfection was detected based on IgM positivity, while the others tested were only IgG positive. Based on this, and in accordance with the high HSV/CMV seroprevalence in comparable Swiss cohorts, we believe that the use of the term reactivation most likely reflects the clinical condition. However, we have limited our use of the word ‘reactivation’ throughout the manuscript, and in many places updated it as ‘herpesvirus disease’ in order to be more accurate. This does not change the overall message of the manuscript.*

7/ The authors might tone down a little bit the conclusion, because many parameters were compared between patients with or without AAB. I doubt the association between AAB and viral reactivation (which makes a lot of sense anyway) would remain statistically significant after accounting for multiple testing. The immunological evidence, paradoxically, may be stronger.

*> We thank the reviewer for this comment. We believe that we have been reasonably cautious with our interpretations throughout the manuscript, and have fairly highlighted some of the limitations of the study design in our discussion. We have noted in the abstract that our statistical testing is adjusted for other factors such as age, gender and systemic steroid treatment, and have tried to remove any overly superlative conclusions throughout.*

**Reviewer #2:**

Busnadiego et al. investigated the interplay between autoantibodies against antiviral type I IFNs, COVID-19, and reactivation of persistent Herpesvirus infections. In about 10% of their over 100 ICU patients with COVID they detected antibodies neutralizing IFN-a2 and/or IFN-o, mostly of the IgG type. The antibodies were present in the plasma as well as in tracheobronchial secretions, indicating they could positively influence SARS-CoV-2 replication in situ. No particular pattern of antibody increase or decrease could be discerned over the observation period. While the association of such anti-IFN antibodies with COVID-19 has already been described, the authors went on to demonstrate that the anti-IFN autoantibodies were also a predictor of Herpes virus (HSV, CMV) reactivation in the COVID patients.

This important and interesting study shows that the association of anti-IFN autoimmunity with severe COVID-19 may not only (or not necessarily) be based on an elevated SARS-CoV-2 replication, but perhaps because IFN neutralization can reactivate latent Herpes virus infections, a known other COVID risk factor. Moreover, the authors have established a convenient and sensitive neutralizing assay for type I IFNs that will facilitate similar screening efforts in the future.

The results are therefore advancing our knowledge, will inspire further studies, and may help to improve diagnosis and treatment of COVID-19. I have only minor remarks.

*> We thank the reviewer for their very positive assessment of our study and its importance.*

Minor points:

-	Apart from the mention that the bead-based assay for autoantibody screening was based on methods from a previous study, the rationale for choosing these three specific IFN subtypes remains unclear.

*> We have now added that these IFNs were taken as representative of different type I IFNs based on the seminal study of Bastard et al., Science, 2020 which had characterized multiple IFNs (line 217).*

-	Patients were also tested for anti-IFN-b autoantibodies (line 202), but the result of the screen is neither shown nor mentioned. Only the lack of a correlation with IFN-a2 antibodies is described later on (line 236). Similar plots as the ones in Fig.1A and B would be helpful to understand the role (or non-role) anti-IFN-b autoimmunity.

*> We did not identify any patients who were positive for anti-IFNbeta autoantibodies. We have now added a sentence to clarify this on lines 242-243. The IFNbeta screening data, as they relate to IFNalpha, are also included as Supplementary Figures 2A & B.*

-	Why are the detection thresholds for IFN-a2 and IFN-o antibodies set differently? Please provide an explanation.

*> This is based on the observed variability of healthy donor plasma reactivity to each IFN (IFNalpha gave much less reactivity than IFNomega, where a greater range of ‘background’ reactivities were observed for healthy donor samples). We therefore decided that we could be more stringent in our cut-off threshold for IFNalpha (10 SDs) so as to more rigorously minimize the possibility of false-positives with this IFN. We have added a sentence to explain this on lines 164-165.*

-	Is it known whether the anti-IFN-a2 antisera would also be neutralizing other IFN-a subtypes? Please add at least a short discussion.

*> We have now added a sentence to discuss this on lines 372-373.*

-	Description of figure 1 in the main text: Please do always provide the number of samples and not only of patients in the text, as it was done in line 219 for the IFN-a2 IgG positives. Otherwise it looks like a discrepancy between the numbers in text and the dots in the figure.

*> We have now added the sample numbers to the relevant places.*

**Reviewer #3:**

Busnadiegoa et al assessed the presence of anti IFN-I ab in severe covid.
They confirmed previous reports and highlight the high prevalence of these AAB in critical COVID-19 partients (around 10%).

They found an association between herpes reactivation and the presence of AAB.
i regret that only a part of patients have been investigated for these analysis.
Moreover, I am not convinced by the direct link between latent virus reactivation and presence of AAB anti IFN-I.

Ex vivo models are requested to deeper investigate the relationship between herpes virus and IFN impairment.

this would greatly improve the quality of the manuscript.

*> We thank the reviewer for their assessment of our study, and agree that further work beyond the scope of this Discovery Report will be important to solidify a direct link between herpesvirus reactivations and anti-IFN autoantibodies. Indeed, we have discussed the study design limitations in our manuscript and proposed that future studies with higher participant numbers, and a pre-defined systematic sampling procedure for the detection of herpesvirus reactivations will be essential. Interestingly, we believe that there are already a number of ex vivo, clinical, and laboratory-based studies that support the idea of a link between type I IFN impairment and herpesvirus reactivations. We have discussed these on lines 437-451.*
